# Retrospective analysis of clinical characteristics and treatment of children and adolescents with depression

**DOI:** 10.3389/fpsyt.2023.1036314

**Published:** 2023-07-27

**Authors:** Xiaolu Jiang, Hongyu Zheng, Rong Yang, Shuo Wang, Hui Zhong

**Affiliations:** ^1^Department of Child and Adolescents, Affiliated Psychological Hospital of Anhui Medical University, Hefei, Anhui, China; ^2^Anhui Mental Health Center, Hefei, Anhui, China; ^3^School of Mental Health and Psychological Sciences, Anhui Medical University, Hefei, Anhui, China; ^4^Department of Child and Adolescents, Fourth People’s Hospital, Hefei, Anhui, China

**Keywords:** depression, childhood, early adolescence, late adolescence, antidepressants

## Abstract

**Objective:**

To analyze the demographic and clinical characteristics and treatment among children and adolescents with depression in different age groups of onset.

**Methods:**

635 children and adolescents with depression in a hospital from January 2014 to December 2021 were collected by e-case, and grouped according to age of onset, including 115 cases in childhood 8-12, 359 cases in early adolescence 13-1 and 161 cases in late adolescence 16-18, and the general conditions, clinical characteristics, and treatment were compared between the three groups.

**Results:**

Females had more onset and were more likely to have psychotic symptoms in childhood, short duration and hospitalization in early adolescence increased year by year, and males had more onset and less hospitalization in late adolescence. There were no statistical differences in medication regimen, suicide, length of hospitalization, or family history between the three groups.

**Conclusion:**

Children and adolescents with depression have their unique clinical characteristics at different age of onset and need to enhance prevention and individualized treatment.

## 1. Introduction

About 3.1% of the total global burden of disease is attributable to mental and psychological disorders (1), with depression accounting for the largest share of the burden of mental illness (2). An epidemiological study in the United States found that the lifetime prevalence of depression in adolescents was 11–14%, and approximately 20% of adolescents experienced severe depression before the age of 18 (3). A domestic study on the prevalence of mental disorders in children and adolescents aged 6–16 found that the overall prevalence of mental disorders is as high as 17.5%. Attention deficit hyperactivity disorder (ADHD), oppositional defiant disorder (ODD), and major depressive disorder (MDD) are the most common mental disorders in children and adolescents, among which the prevalence of the depressive disorder was 3.0% (4). In addition, a longitudinal study of adolescents whose parents had a history of mood disorders found that depression was the strongest predictor of suicidal behavior in adolescents and young adults. The study also found that children with depression were almost three times more likely to commit suicide before adolescence than adolescents with depression (5, 6). Notably, the female gender and suicidal ideation are predictors of suicidal behavior in adolescents (6).Adolescence (ages 10–19) is a unique formative period with multiple physical, emotional, and psychological factors, and social change is critical for the development and maintenance of social and emotional habits. Promoting mental health and preventing adverse experiences and risk factors that may affect the growth potential are important aspects of the physical and mental health of adolescents and adults. Illness is a complex and multifaceted process influenced by many biological and environmental factors. Numerous studies have confirmed the impact of cognitive, psychosocial, and biological developmental levels on the susceptibility of children and adolescents to MDD. However, it remains difficult to reach a consensus on the psychopathology of depression in children and adolescents, the incidence of depressive symptoms, and other relevant phenomenological differences.

Thirty years ago, depression was thought to be an adult-only mental illness, with children and adolescents being “immune” to it, considering their low mood or their depressive manifestations as a “normal” part of growing up. Existing research shows that children and adolescents can suffer from depression as well as, and that depression in children and adolescents can lead to a range of adverse outcomes, such as suicide, impaired social functioning, lower educational attainment, and possible later physical and mental illness (7). Adolescence is a risk factor for depressive episodes with a high recurrence rate and poor functional prognosis. After entering adolescence, people are emotionally vulnerable, easily depressed when they experience setbacks, and have a growing sense of independence from their parents, being more sensitive and prone to depressive symptoms. Compared to children, adolescents have more anhedonia, narcolepsy, and difficulty concentrating, while feelings of worthlessness are more common in children (8, 9). Symptoms of depression in children and adolescents differ from those in adults. They show fewer core symptoms of depression and often have more problems with behavior, school, and interpersonal relationships. It is often accompanied by behavioral problems, such as Internet addiction, violent conflict, and self-injury. It seriously affects the quality of life of young people and places a heavy burden on their families and society. In a study of 276 adult patients with relapsing depression, approximately 50% had an onset before the age of 18 years (10). Individuals with depressive episodes in childhood or adolescence had a higher risk of suicide, with 48% of those with early onset depression attempting suicide compared to 26% of those with adult depression. This highlights the importance of the appropriate and effective management of depression in children and adolescents. However, the current diagnosis of depression in children and adolescents is still based on diagnostic criteria for adults. Therefore, to promote the comprehensive recovery of adolescent depression patients, it is important to study the clinical characteristics of adolescent depression patients.

In this study, growth stages were divided into childhood (8–12 years old), early adolescence (13–15 years old), and late adolescence (16–18 years old). The main purpose of this study was to better understand the demographic and clinical characteristics of depression at different onset ages from childhood to late adolescence, to improve family and social awareness of depression in children and adolescents, and to provide clinical evidence for targeted assessments and interventions.

## 2. Materials and methods

Two trained postgraduate psychiatry students with depression in children and adolescents aged ≤18 years hospitalized at the Fourth People’s Hospital in Hefei, China, from January 2014 to December 2021 were included as study subjects *via* electronic medical records. Inclusion criteria were: The inclusion criteria were: (1) they met the diagnostic criteria for depression according to the Inter-national Classification of Diseases and Diagnostic Criteria (ICD-10); (2) the medical records were complete and reliable, and no study had a lack of data and information. The exclusion criteria included: (1) schizophrenia, bipolar disorder, intellectual disability, or other mental disorders; (2) alcohol and drug use; and (3) neurological diseases or organic brain damage. A total of 635 subjects were included in this study, consisting of 456 females and 179 males, aged 11–18 years (15.33 ± 1.71) years old.

Data on the general condition and clinical characteristics of inpatients were collected from the electronic records of the hospital. (1) The general situation investigation included sex, age, family history of mental disorders, physical diseases. (2) Clinical feature data collection. All patients were clinically assessed within 24 h after admission by staff trained in the use of HAMA (11) and HAMD-24 scales (12) (Cronbach’s alpha =0.8), including diagnosis, treatment, suicidal behavior, psychotic symptoms, HAMA score and HAMD score. A total of 14 HAMA items are scored at 5 levels ranging from 0 to 4. The sum of scores of each item is the total score. The higher the score is, the more serious the degree of anxiety is. Most of the items in HAMD-24 were graded from 0 to 4, and a few items were graded from 0 to 2, and graded from 3. The sum of the scores of all items was the total score. The higher the score, the more serious the depression, and the total score > 8 indicated depression. Suicidal behavior assessment: The medical record clearly shows “suicidal behavior” and gives a detailed description of the method of suicide. (3) The treatment situation was according to the medical record and doctor’s order data. The medication regimen was collected on the day of discharge according to data entry and drug treatment regimens were divided into antidepressants alone, antidepressants + antipsychotics, antidepressants + antipsychotics + mood stabilizers, and antidepressants + mood stabilizers. The data was gathered and proofread by multiple people. Based on the age of onset, the cases were divided into children group (8–12), early adolescence group (13–15) and late adolescence group (16–18).

SPSS 26. 0 was used for data processing and analysis. All continuous variables were expressed as mean ± standard deviation, and skewed measures were expressed as median and quartiles, and subjects were compared between the three groups using one-way analysis of variance (ANOVA) for demographic and HAMD scores. The chi-square test was used to compare differences between groups, and Bonferroni’s method was used for multiple comparisons. *p* < 0.05 (two-tailed) indicates that the differences are statistically significant.

## 3. Results

### 3.1. Demographic and clinical characteristics of the study population

**Figure 1:**
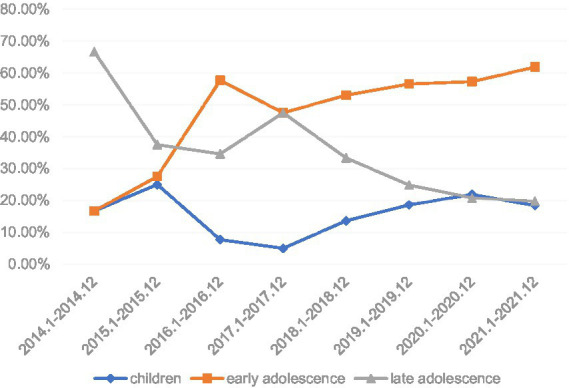
Incidence trends in different age stages of hospitalized children and adolescents in the past 8 years.

A total of 635 participants were included, including 115 in the childhood group (18.1%), 359 (56.5%) in the early adolescence group, and 161 (25.4%) in the late adolescence group. Basic demographic information and clinical characteristics of the patients are shown in Table 1. Among them, 28.2% were men, and 71.8% were women. The mean age at admission was 15.33 ± 1.71 years. There were 494 (77.8%) patients with first onset, 159 (25.0%) patients with psychotic symptoms, with an average age of onset of 14.21 ± 1.87 years, and an average disease duration of 14.54 ± 13.57 months. The Hamilton Depressing Rating Scale (HAMD) and Hamilton Anxiety Rating Scale (HAMA) total scores at admission were 21.42 ± 9.15 and 12.54 ± 6.45, respectively. As time progressed (Figure 1), the number of hospitalizations for depressive disorder increased yearly, the proportion of hospitalizations in the child group increased compared with previous years, and the proportion of hospitalizations in the late adolescence group tended to decline (*X*^2^ = 29.736, *p* < 0.01).

**Table 1:** Basic demographic information and clinical characteristics of children and adolescents with depression.

	*N* (%)
Demographic variables	
Male	179(28.2%)
Age at admission, years	15.33 ± 1.71
Clinical characteristics	
Days of hospitalization	36.74 ± 23.02
First onset	494(77.8%)
Age of onset, years	14.21 ± 1.87
8 ≤ age ≤ 13	115(18.1%)
13 ≤ age ≤ 18	520(81.9)
Duration of illness, months	14.54 ± 13.57
Major depression with psychotic symptoms	159(25.0%)
HAMA total at admission	12.54 ± 6.45
HAMD total at admission	21.42 ± 9.15

### 3.2. Comparison of general data and distribution of hospitalizations

By ANOVA comparison, we found no statistically significant differences between the three groups in terms of length of stay, family history of psychiatric disorder, suicidal behavior, and total HAMD24 score (all *p* > 0.05), while significant differences were found in sex and course of disease among (all *p* < 0.05) (Table 2). Among them, women in the childhood group and men in the late adolescence group had a higher incidence, and the disease duration in the early adolescence group was shorter than that in the other two groups. In terms of clinical characteristics, there were statistically significant differences in psychotic symptoms among the three groups, among which the children were more likely to have psychotic symptoms.

**Table 2:** Comparison of general information between the three groups.

	children (*n* = 115)	early adolescence (*n* = 359)	late adolescence (*n* = 161)	*Z*/*χ*2/F	*p*
Gender					
Male	16	105	58	16.660	<0.001**
Female	99	254	103		
Family history of mental illness					
positive	7	19	8	0.172	0.918
Negative	108	340	153		
Psychotic symptoms					
With	38	89	32	6.224	<0.05**
without	77	270	129		
Suicide					
With	19	77	23	4.199	0.123
without	96	282	138		
Days of hospitalization	36.39 ± 21.99	36.51 ± 23.16	37.49 ± 23.53	0.117	0.890
HAMD_24_	22.59 ± 8.06	21.09 ± 9.29	21.25 ± 9.58	1.202	0.301
Duration of illness	15.00(12.00，36.00)	12.00(6.00，24.00)	6.00(2.50，12.00)	84.617	<0.001**

HAMD, Hamilton Depression Scale, *p < 0.05; **p < 0.001.

### 3.3. Types of medicines on discharge

The discharge prescriptions of different antidepressants and discharge regimens of the 635 patients were included in the statistical analysis. Of these, 156 patients were on monotherapy and 479 were on combination therapy. The top five antidepressants were sertraline in 399 cases (52.2%), duloxetine in 114 cases (18.0%), mirtazapine in 95 cases (12.4%), escitalopram in 51 cases (8.0%), and venlafaxine in 50 cases (7.9%) (Table 3). Comparing the frequency of commonly used antidepressants and treatment regimens in patients of different age groups, it was found that among the top five antidepressants, there was no significant difference in the frequency of antidepressant use in hospitalized patients of different ages (all *p* > 0.05; *X*2 = 8.082, *p* = 0.425) (Table 4). There were no significant differences in depressive drugs, antidepressant drug + antipsychotic drug combination, antidepressant drug + antipsychotic drug + emotion stabilizer combination, and other treatments (all *p* > 0.05) (Table 5).

**Table 3:** Discharge prescriptions for different antidepressants.

	*N* (%)	Dosage (mg/d)	Dosage range (mg/d)
Escitalopram	51 (8.0%)	15 ± 4.58	5.00–20.00
Fluvoxamine	18 (2.8%)	176.39 ± 17.05	20.00–120.00
Fluoxetine	26 (4.1%)	43.08 ± 15.69	20.00–60.00
paroxetine	12 (1.9%)	35 ± 13.14	10.00–60.00
Sertraline	399 (62.8%)	119.76 ± 49.47	25.00–120.00
Mirtazone	95 (15.0%)	18.95 ± 11.93	7.50–60.00
Venlafaxine	50 (7.9%)	175.98 ± 48.98	75.00–225.00
Duloxetine	114 (18.0%)	55.58 ± 17.05	20.00–120.00

Data were expressed by *N* (%) or mean ± standard deviation.

**Table 4:** Frequency of common antidepressant use and treatment regimen in patients of different ages.

	Sertraline	Duloxetine	Mirtazone	Venlafaxine	Escitalopram	*χ*2	*p*
children (*n* = 115)	90 (78.3)	17 (14.8)	14 (12.2)	7 (6.1)	9 (7.8)	8.082	0.425
early adolescence (*n* = 359)	227 (63.2)	68 (18.9)	54 (15.0)	30 (8.4)	27 (7.5)		
late adolescence (*n* = 161)	82 (51.0)	29 (18.0)	24 (15.0)	13 (8.1)	15 (9.3)		

*p < 0.05; **p < 0.001.

**Table 5:** Comparison of the treatment of depression in childhood, early adolescents and late adolescents.

	medical treatement				*χ*2	*p*
	A	B	C	D		
children (*n* = 115)	30	58	21	6	10.024	0.124
early adolescence (*n* = 359)	80	160	70	49		
late adolescence (*n* = 161)	46	61	32	22		

A: Antidepressants; B Antidepressants + Antipsychotics; C: Antidepressant + Antipsychotic + Mood Stabilizer treatment; D: Antidepressant + Mood Stabilizer treatment. Data were expressed by *N* (%) or mean ± standard deviation; statistic values were expressed by Pearson’s *χ*2 tests. *p < 0.05; **p < 0.01.

## 4. Discussion

Reviewing the demographics, clinical characteristics, and drug treatment plans for children and adolescents with depression in different age groups from 2014 to 2021, results showed that women in the children group had more symptoms and psychotic symptoms, and the disease duration in the early adolescence group was shorter than that in the other two groups. In the late adolescence group, males had more morbidities and fewer psychotic symptoms. As time progressed, the number of hospitalizations for depressive disorder increased yearly, the proportion of hospitalizations in the child group increased compared with previous years, and the proportion of hospitalizations in the late adolescence group tended to decline. There were no statistically significant differences in the frequency of antidepressant use or treatment regimens among the three groups.

Our study found that in childhood and adolescent depression, gender differences in depression appeared in childhood versus late adolescence. The onset was more frequent in females in childhood and more common in males in late adolescence. Regarding gender differences, the results of a 1998 study showed that gender differences in depression appeared in early adolescence (around 12–13 years old) and mid-adolescence. Angold et al. used the Children’s Depression Inventory (CDI) to evaluate depressive symptoms. The results showed that gender differences in depressive symptoms in children and adolescents aged 9–16 years in the UK appeared at 13 years of age. Previously, there was no statistically significant difference in the detection rate of depressive symptoms between boys and girls. In addition, another study showed that, in the general population, the incidence of depression in boys during childhood did not differ by sex and was even slightly higher than that of girls (13). Female predominance in depression is thought to appear at 13–15 years of age (14). However, this differs from our findings. The age of onset in women in this study was more commonly 8–12 years. A meta-analysis of nearly a decade of studies on adolescent depression found that negative life events in early female adolescents can increase the risk of depression, most likely by increasing the individual’s sensitivity to and amplifying stress, and other pathways increase adolescents’ susceptibility to depression (15). Furthermore, some theorists believe that physiological changes during puberty increase the risk of depression in girls (16). The exact timing of depression may indicate which physiological changes may increase the risk of depression in girls. Social changes, such as school transitions, improved living standards, and changes in parental divorce rates may be responsible for gender differences in depression key turning points. In conclusion, although gender differences in depression symptoms in adolescence have not yet been unified, research generally supports the phenomenon of “female dominance” in depression symptoms in adolescence and considers that gender differences in sex hormone levels in adolescence are the main reasons. Whereas males are more likely to experience depression as they transition from early to late adolescence, on the one hand, there is a stronger association between depressive problems and poor academic performance in boys compared to girls (17), and poor academic performance may lead to negative feedback from parents, peers, teachers, and others. In turn, this negative feedback may trigger negative self-perceptions, which in turn trigger a vicious cycle of negative emotions. On the other hand, it is possible that because boys are more active and naughty than girls in this age group, and thus are generally subjected to higher levels of criticism and chastisement at school than girls, they are also more psychologically stressed than girls and therefore tend to show more anxiety and anxiety and sadness, and therefore boys are at higher risk of developing depressive symptoms than girls (18).

Depression can be divided into two subtypes: depression with and without psychotic symptoms. Depression with psychotic symptoms refers to meeting the diagnostic criteria for depression, and is accompanied by hallucinations, delusions, depressive stupor, and other symptoms. Our study found that 25.0% (159 cases) of depressive children and adolescents had psychotic symptoms. Ryan et al. found that the prevalence of psychotic features in outpatients with major depressive disorder was 18% (19), while Haley et al. showed a prevalence of 45% in a sample of hospitalized adolescent patients (20). Some studies have shown that the proportion of depression with psychotic symptoms in patients with depression is 15–19% (21), which may be related to differences in the study population, ethnicity, assessment of psychotic symptoms, and diagnostic criteria. In terms of associated psychiatric symptoms, the age of onset is younger and more common in childhood, consistent with previous studies (22). A study of 129 depressed adolescents found that individuals with psychotic symptoms were more likely to have a history of childhood trauma, especially severe sexual abuse (23). Previous studies have also shown that patients with adverse childhood events have an earlier age of onset than patients without (24), indicating that adverse childhood life events may lead to an earlier onset and the chronicity of depression in patients with depression, obvious social function impairment, and other characteristics. McGee et al. found that healthy children who had hallucinatory experiences before the age of 11 were more likely to develop more severe depression (25). Further major depressive disorder with psychotic symptoms is associated with more severe symptoms, worse prognosis (26), greater risk of relapse (27, 28), and higher mortality rates (28). Therefore, childhood and adolescence are critical periods for the prevention of and early intervention for depression. Reasonable intervention can reduce or delay the related health problems caused by depression, whether it is a normal group or a sick child, such as hallucinations, delusions, and other abnormal experiences. Thus, early detection should be desirable. In clinical practice, it is difficult for clinicians to collect mental symptoms of sick children because patients with psychotic symptoms are often reluctant to mention their abnormal perception and thinking due to embarrassment, which requires clinicians and the use of flexible and proficient communication skills to define whether an affected child has psychotic symptoms.

Compared with the childhood and late adolescence groups, the early adolescence group had a shorter course of disease, and the proportion of hospitalizations increased yearly. On the one hand, this may be due to people paying more attention to children aged 13–15 than other age groups, probably being the main reason for family members and patients seeking medical treatment as soon as possible. On the other hand, it may be due to the fact that early adolescents are in a critical period of physical and mental development. During the process of aging, their body structure changes significantly, while their psychological and physiological development is not mature, living through a contradiction between naivety and maturity, dependence and independence. At this psychologically sensitive and fragile stage, it is easy to take a one-sided and extreme view of problems, to be unable to properly handle complex interpersonal relationships and stressful events, and often be accompanied by some physical discomfort symptoms; the physical condition becomes worse, and it is easier for individuals to cast their eyes on themselves, while ignoring other aspects. The mentality is further deteriorated, resulting in the accumulation of bad emotions, eventually developing into a depressive disorder. Previous research has found an association between peer bullying and depression (29). The mechanism may be that peer bullying as a chronic stressor leads to hypervigilance, followed by learned helplessness, and finally, depression (30). In addition, study pressure is an important factor related to depression. The mechanism may be that under external pressure, the individual’s hypothalamic–pituitary–adrenal axis neuroendocrine system is disorderly regulated, and a large amount of stress hormones are released, resulting in damage to the brain regions related to emotion (31). This may be an important factor in the occurrence of depression among children and adolescents.

The present study showed no statistical difference in the choice of antidepressants as well as treatment regimens among the three groups of depressed patients of different ages, and internationally, most treatment guidelines for children and adolescents recommend psychotherapy for mild depressive episodes and a combination of psychotherapy and antidepressants for moderate to severe depressive episodes (31). The top 5 antidepressants for children and adolescents in this study were sertraline, duloxetine, mirtazapine, escitalopram, and desvenlafaxine, in that order. In the United States, only two antidepressants are approved for use in children and adolescents: fluoxetine is approved for MDD in children, and both fluoxetine and escitalopram are approved for use in adolescents (FDA, accessed 2021a). However, other studies have shown that sertraline and citalopram have some efficacy and fair safety in depression in children and adolescents and can be used as second-line treatment (32, 33). Sertraline, as a novel antidepressant, efficiently inhibits the reuptake of 5-hydroxytryptamine in the central nervous system and regulates norepinephrine, and is used in the clinical treatment of patients with various depressive and obsessive–compulsive disorders. Especially in children and adolescents, patients can gradually see effects and significant improvement in their condition after about 3–4 weeks of using the drug until they return to normal (34). Escitalopram belongs to the class of SSRIs and is the active S-isomer of citalopram, with better selectivity and inhibition of 5-HT reuptake than citalopram, with stronger pharmacological effects and fewer adverse effects. Numerous studies have confirmed the higher remission rate of escitalopram than placebo in the treatment of adolescent depression (35–37). In addition, duloxetine and desvenlafaxine were approved by the FDA in 2004 and 2008, respectively, as SNRIs for the treatment of depression in adults. Although some studies have found that venlafaxine and duloxetine are poorly tolerated in depression in children and adolescents. However, in terms of efficacy, duloxetine and venlafaxine were significantly better than placebo (38). Currently, both duloxetine and venlafaxine are used beyond the instructions for the treatment of depression in children and adolescents (39); therefore, the efficacy of duloxetine and desvenlafaxine in the treatment of depression in children and adolescents needs to be evaluated in further studies, especially in children and adolescents with poorly tolerated depression. In addition, this study found differences in clinical characteristics among the three stages of depression in children and adolescents without differences in medication regimens, and the selection of antidepressants should be based on the patient’s gender, age, family history, duration of illness, symptom profile, severity of illness, co-morbidities, and physical illness status.

It has been suggested by psychologists that “the fortunate are cured by childhood throughout their lives, and the unlucky are cured by childhood throughout their lives.” As the onset of depressive disorders is gradually becoming younger, depressive symptoms are already very common in children during their childhood. Our patients should enjoy the fun of childhood and experience the joy and worries of growing up at this age, but for various reasons they suffer from mood disorders, which seriously affect their learning and normal life, and suffer from great pain for this reason. Therefore, it is important to study depression in children and adolescents. Our study found different clinical characteristics of children and adolescents with depression in different age groups, so that subsequent clinicians can develop more appropriate treatment plans based on this study and improve the prognosis of the affected children.

There are some limitations in this study: firstly, the sample size was rather small and limited to the Hefei Fourth People’s Hospital. Second, the selected patients’ depression levels are the scores of the initial assessment of previous inpatients, which may be influenced by the patients’ recent experiences and may not reflect their long-term depression levels sufficiently; third, in comparing “childhood,” “early adolescence” and “late adolescence,” we only used age to distinguish these three samples. Third, in comparing “childhood,” “early adolescence,” and “late adolescence,” we used only age to distinguish between the three samples, describing the degree of difference between the three samples rather than trends.

## 5. Conclusion

In summary, the effects of depression, a common psychiatric disorder in children and adolescents, usually persist into adulthood and may also cause or exacerbate physical and/or other mental health problems. Therefore, addressing the heavy burden of adolescent depression on individuals and families has significant public health implications. Our findings revealed different clinical characteristics of children and adolescents with depression at different times, thus further research on the clinical characteristics of children and adolescents with depression at different ages is necessary to develop better treatment plans.

## Data availability statement

The original contributions presented in the study are included in the article/supplementary material, further inquiries can be directed to the corresponding author.

## Ethics statement

The studies involving human participants were reviewed and approved by Ethics Committee of the Fourth People’s Hospital of Hefei, Anhui, China. Written informed consent for participation was not required for this study in accordance with the national legislation and the institutional requirements.

## Author contributions

XJ, HoZ, RY, and SW data collection. XJ conceptualization and draft writing. HuZ provided financial support. All authors contributed to the article and approved the submitted version.

## Funding

This work was supported by the National Key Research and Development Program of China (no 2018YFC1314300), the National Natural Science Foundation of China (no 32071020) and Hefei Key Specialties (no Hwk2019yb0022).

## Conflict of interest

The authors declare that the research was conducted in the absence of any commercial or financial relationships that could be construed as a potential conflict of interest.

## Publisher’s note

All claims expressed in this article are solely those of the authors and do not necessarily represent those of their affiliated organizations, or those of the publisher, the editors and the reviewers. Any product that may be evaluated in this article, or claim that may be made by its manufacturer, is not guaranteed or endorsed by the publisher.
